# Silencing of Histone Deacetylase 6 Decreases Cellular Malignancy and Contributes to Primary Cilium Restoration, Epithelial-to-Mesenchymal Transition Reversion, and Autophagy Inhibition in Glioblastoma Cell Lines

**DOI:** 10.3390/biology10060467

**Published:** 2021-05-26

**Authors:** Alejandro Urdiciain, Elena Erausquin, María V. Zelaya, Idoya Zazpe, José L. Lanciego, Bárbara Meléndez, Juan A. Rey, Miguel A. Idoate, Natalia A. Riobo-Del Galdo, Javier S. Castresana

**Affiliations:** 1 Department of Biochemistry and Genetics, University of Navarra School of Sciences, 31008 Pamplona, Spain; aurdiciain@alumni.unav.es (A.U.); eerausquin@alumni.unav.es (E.E.); 2 Department of Pathology, Hospital Complex of Navarra, 31008 Pamplona, Spain; mv.zelaya.huerta@navarra.es; 3 Department of Neurosurgery, Hospital Complex of Navarra, 31008 Pamplona, Spain; idoya.zazpe.cenoz@navarra.es; 4 Neurosciences Division, Center for Applied Medical Research (CIMA), University of Navarra, 31008 Pamplona, Spain; jlanciego@unav.es; 5 Molecular Pathology Research Unit, Virgen de la Salud Hospital, 45005 Toledo, Spain; bmelendez@sescam.jccm.es; 6 IdiPaz Research Unit, La Paz University Hospital, 28046 Madrid, Spain; jreyh@salud.madrid.org; 7 Department of Pathology, University of Navarra Clinic, 31008 Pamplona, Spain; maidoate@unav.es; 8 School of Molecular and Cellular Biology, Faculty of Biological Sciences, University of Leeds, Leeds LS2 9JT, UK; n.a.riobo-delgaldo@leeds.ac.uk; 9 Leeds Institute of Medical Research, Faculty of Medicine and Health, University of Leeds, Leeds LS2 9JT, UK; 10 Leeds Cancer Research Centre, University of Leeds, Leeds LS2 9JT, UK

**Keywords:** HDAC6, siRNA, glioblastoma, epithelial-to-mesenchymal transition, primary cilium, autophagy, sonic hedgehog

## Abstract

**Simple Summary:**

Glioblastoma multiforme (GBM) is the most common as well as the most aggressive malignant brain tumor, with an overall survival of almost 15 months. Histone deacetylase 6 (HDAC6), an enzyme related to the deacetylation of α-tubulin, is overexpressed in GBM. The aim of our research was to study the effects of HDAC6 silencing in GBM cells. We first confirmed the overexpression of HDAC6 in GBM tissue (*n* = 40) against control brain (*n* = 10). Treatment with siHDAC6 diminished viability, clonogenic potential, and migration ability in GBM-derived cell lines. HDAC6 inhibition also reverted the mesenchymal phenotype, inhibited the Sonic Hedgehog pathway, restored primary cilium structure, and decreased autophagy. Thus, we confirm that HDAC6 is a good therapeutic target for GBM treatment.

**Abstract:**

Glioblastoma multiforme, the most common type of malignant brain tumor as well as the most aggressive one, lacks an effective therapy. Glioblastoma presents overexpression of mesenchymal markers Snail, Slug, and N-Cadherin and of the autophagic marker p62. Glioblastoma cell lines also present increased autophagy, overexpression of mesenchymal markers, Shh pathway activation, and lack of primary cilia. In this study, we aimed to evaluate the role of HDAC6 in the pathogenesis of glioblastoma, as HDAC6 is the most overexpressed of all HDACs isoforms in this tumor. We treated glioblastoma cell lines with siHDAC6. HDAC6 silencing inhibited proliferation, migration, and clonogenicity of glioblastoma cell lines. They also reversed the mesenchymal phenotype, decreased autophagy, inhibited Shh pathway, and recovered the expression of primary cilia in glioblastoma cell lines. These results demonstrate that HDAC6 might be a good target for glioblastoma treatment.

## 1. Introduction

Glioblastoma (GBM), or grade IV astrocytoma, is the most common form of malignant brain tumor as well as the most aggressive one. It corresponds to 14.6% of all brain tumors, 48.3% of malignant brain tumors, and 57.3% of gliomas, making GBM the third most common brain tumor [1]. Current treatment of GBM consists of maximal resection followed by radiotherapy and chemotherapy [2]. Despite this therapeutic effort, overall survival of GBM patients five years after diagnosis is 6.8% [1], which highlights the need of research for new therapeutic targets against this kind of tumor. 

Histone deacetylase 6 (HDAC6) is a IIb class of HDACs and is the isoform that presents the highest expression in GBM [3]. This protein regulates biological processes, such as migration and unfolded protein response. HDAC6 is able to deacetylase non-histone substrates, like cortactin, HSP90, or acetylated α-tubulin, with the latter being its main target [4,5]. Acetylated α-tubulin takes part in the structure of the primary cilium, which has been seen to be lost in GBM cells [6,7]. The primary cilium is essential for several signalling pathways, such as Sonic Hedgehog, PDGFR, and Notch [8,9,10,11]. Loss of this structure can be seen in other tumors, like lung [12], prostate [13], and ovarian [14] cancer. 

Epithelial-to-mesenchymal transition (EMT) is a cellular programme by which epithelial cells lose the expression of epithelial markers and acquire the expression of mesenchymal markers, increasing its migration capacity and its resistance to apoptosis. This mesenchymal state can be reverted in a process called mesenchymal-to-epithelial transition (MET). Epithelial-derived tumors have been seen to be the most lethal, such as breast, colon, pancreas, and kidney cancer [15]. EMT can be reverted by treatment with inhibitors of HDACs [16], which define HDACs as new therapeutic targets against cancer [17,18,19]. 

Autophagy is a process by which cellular components are degraded to maintain the cellular homeostasis and protect cells from metabolic stress. Autophagy provides amino acids and free fatty acids to the cells to obtain energy from tricarboxylic acid cycle (TCA) [20]. The autophagic process is stimulated in cancer cells and supplies the higher demands of energy that these cells need to proliferate, which may centre autophagy as a therapeutic target for cancer. 

In this study, we aimed to know the effects of silencing HDAC6 in GBM cell lines. HDAC6 expression associated to clonogenicity, cell migration, and cell proliferation and also to EMT, Shh pathway activation, lack of a primary cilium, and to autophagy promotion. Our HDAC6 siRNA-silencing experiments demonstrate a reversal of all those cellular characteristics, making of HDAC6 a potential therapeutic target for GBM treatment.

## 2. Materials and Methods

### 2.1. Patient Samples

Frozen sections of control brains (temporal lobe: *n* = 7, parietal lobe: *n* = 7) and of GBM samples (*n* = 11) were a kind gift from Dr. Lanciego and Dr. Idoate, respectively, both coauthors of the study. Proteins were extracted with RIPA buffer, and RNA was extracted with TRIzol reagent with the help of an Ultra turrax (Janke & Kunkel, Ika Labortechnik, Staufen, Germany). The present study was authorized by the Ethics Committee of the University of Navarra (approval no. CEI0502012). Patients provided informed consent for the use of their samples for research. All samples were fully anonymized prior to accessing. 

### 2.2. Immunohistochemistry

In order to compare the expression status of HDAC6 and the autophagic marker p62 between normal human brain tissue (*n* = 10) and GBM tissue (*n* = 40), we performed immunohistochemistry for these proteins at the Department of Pathology of the Hospital Complex of Navarra, Pamplona, Spain (Dr. Zelaya, coauthor of the study). Antibodies used in this experiment were: HDAC6 (1677558S) from Cell Signaling (Danvers, MA, USA), and the p62 antibody was a kind gift from the Hospital Complex of Navarra. 

### 2.3. Cell Culture

Three human GBM cell lines were used for the study: LN405 was purchased from DSMZ (Braunschweig, Germany), T98G was obtained from the European Collection of Authenticated Cell Cultures (Salisbury, UK), and U87MG was obtained from ATCC (Manassas, VA, USA). Provenance of the cell lines is as follows: LN405 (DSMZ ACC 189) [21] are glioblastoma cells established from an astrocytoma tumor (grade IV, glioblastoma) of a 62-year-old woman; T98G (ECACC 92090213) [22] was derived from a glioblastoma multiform tumor from a 61-year-old Caucasian male; U87MG (ATCC HTB14) [23] are brain glioblastoma cells from a male of unknown age; and NHA (Lonza CC-2565) [24] come from normal human astrocytes obtained from brain and spinal cord.

LN405 and T98G cell lines present mutations in PTEN and TP53 genes, while U87MG cell line is mutated in PTEN but has a TP53 wild-type gene [25]. The normal human astrocyte cell line NHA was purchased from Lonza (Walkersville, MD, USA). LN405 and T98G were cultured in Roswell Park Memorial Institute (RPMI) 1640 L-GlutaMAX^™^ and supplemented with 10% fetal bovine serum (FBS) and 1% penicillin/streptomycin (P/S). U87MG was cultured in Dulbecco’s Modified Eagle Medium (DMEM) GlutaMAX^™^, supplemented with 10% FBS, 1% P/S, and 4% non-essential amino acids (NEAA). NHA cell lines were cultured in AGM™ BulletKit™ (Astrocyte Growth Medium, ref. CC-3187, Lonza, Basel, Switzerland) and supplemented with AGM™ SingleQuots™ (ref. CC-4123, Lonza, Basel, Switzerland).

### 2.4. Transfection

GBM cell lines were seeded in 6-well plates at a confluence of 150,000 cells/well. At 24 h post-seeding, cells were transfected with a mix of two different siHDAC6 (SASI_Hs01_00048982 and SASI_Hs02_00340796, both of them purchased from Sigma, St. Louis, MI, USA) following lipofectamine 2000 protocol. MISSION^®^ siRNA Universal Negative Control #1 (SIC001, Sigma, St. Louis, MI, USA) was transfected as a control group. At 72 h post-transfection, cells were harvested and seeded for other experiments, or proteins and RNA were extracted with RIPA buffer and TRIzol reagent, respectively. 

NHA cell line was seeded in 6-well plates at a final confluence of 300,000 cells/well. The following day, cells were transfected following lipofectamine 2000 protocol. Plasmids transfected were: pcDNA3 LIC cloning vector 6A (#30124) and pcDNA-HDAC6-FLAG (#30482); both of them were acquired from AddGene (Watertown, MA, USA).

### 2.5. Viability Assay

Transfected cells were plated in 96-well plates at a final confluence of 2500 cells/well. At 24 h post-plating, medium was changed with fresh medium. In order to know the starting viability, we added MTT (3-(4,5-Dimethyl-2-thiazolyl)-2,5-diphenyl-2H-tetrazolium bromide) reagent at a final concentration of 0.5 mg/mL and incubated for 90 min. After that, cells were washed with ice cold PBS, and 100 µL of DMSO were added to each well. Absorbance was measured at 550 nm in a Multiskan EX (Thermo Electron Corporation, Rockford, IL, USA). The same procedure was done 72 h after medium change. 

### 2.6. Colony Formation Assay

Transfected cells were plated in 6-well plates at a final confluence of 300 cells/well and were incubated for 10 days at 37 °C 5% CO_2_. After that, cells were washed with ice-cold PBS and fixed with 4% paraformaldehyde for 40 min. Fixed cells were then stained with crystal violet for 15 min. The following day, cells were counted using Colony Counter 560 (Suntex, Bocairent, Valencia, Spain).

### 2.7. Wound Healing Assay

siRNA-treated cells were seeded in 24-well plates at a final confluence of 250,000 cells/well for LN405 and T98G and 150,000 cells for U87MG. At 24 h post-seeding, a scratch was done with a 200 µL pipette tip, and cell culture medium was changed out for new medium with 2.5% FBS to halt the proliferation of the cells during the scratch wound healing assay. Photographs were taken at times 0, 24, and 48 h post-scratch. Closure of the scratch was then analyzed with ImageJ software. 

### 2.8. RT-qPCR

Total RNA was extracted from untreated cell lines, or siRNAs-treated GBM cell lines, following TRIzol reagent protocol. cDNA was obtained from 2 µg of RNA using SuperScript ™ II Reverse Transcriptase (Cat. No. 18064, Invitrogen, Carlsbad, CA, USA). RT-qPCR was performed in an iQ5 Multicolor real-time PCR detection system (Bio-Rad Laboratories, Hercules, CA, USA). The sequence and Tm of the different primers is described in Table 1. Data were analyzed following the 2^−^^ΔΔ^^Ct^ method.

### 2.9. Western Blot

Proteins were extracted from the untreated cell lines, from the siRNA-treated GBM cell lines, and from the plasmid-treated NHA cell line. Next, 20 µg of proteins were loaded in each well of an SDS-PAGE, immunoblotted, and incubated overnight with primary antibody: HDAC6 1:1000 (7612, Cell Signaling, Danvers, MA, USA), N-cadherin 1:1000 (13116, Cell Signaling, Danvers, MA, USA), Snail 1:1000 (3879, Cell Signaling, Danvers, MA, USA), Slug 1:1000 (9585, Cell Signaling, Danvers, MA, USA), LC3B 1:1000 (3868, Cell Signaling, Danvers, MA, USA), acetylated α-tubulin 1:10,000 (T6793, Sigma, St. Louis, MI, USA), α-tubulin 1:10,000 (T6074, Sigma, St. Louis, MI, USA), and β-actin 1:10,000 (A5441, Sigma, St. Louis, MI, USA). Secondary antibodies were: α-mouse 1:20,000 (10196124, Fisher scientific, Madrid, Spain) and α-rabbit 1:2000 (10794347, Fisher scientific, Madrid, Spain).

### 2.10. Luciferase Assay

Cells were seeded in 24-well plates at a final confluence of 50,000 cells/well. The following day, cells were transfected with p8Xgli-luciferase (wild type or mutated), pRLTK, and control plasmid or pGli1 at a ratio 4:1:5 in 0.5 µg following lipofectamine 2000 protocol. Two days after transfection, media were changed out for one with 0.5% FBS, and 24 h later, luminescence were measured using Dual Glo luciferase assay system (E2920, Promega, Madison, WI, USA) in a Glomax 20/20 luminometer (Promega, Madison, WI, USA). Gli-luciferase activity was measured in U87MG after siRNA treatment following the same protocol but changing the control or Gli plasmid by 25 pmol of siCTRL or siHDAC6.

### 2.11. Immunofluorescence

Cell lines were seeded in 24-well plates at a confluence of 50,000 cells/well. The well contained a sterilized coverslip. At 48 h post-seeding, medium was changed out for one without FBS, and, the following day, cells were fixed with 4% paraformaldehyde for 10 min. After that, cells were permeabilized with 0.025 Triton-X100 in PBS and blocked with blocking solution (0.1 PBS-T 0.3M glycine 10% Normal Goat Serum) for 1 h. Antibodies were incubated overnight: acetylated α-tubulin 1:500 (T6793, Sigma, St. Louis, MI, USA) and γ-tubulin 1:500 (T3559, Sigma, St. Louis, MI, USA). Cells were washed three times with PBS, 5 min each wash, and then were incubated with secondary antibody: α-mouse AlexaFluor^®^ 594 1:500 (A21203, Thermo Fisher, Waltham, MA, USA) and α-rabbit AlexaFluor^®^ 488 1:500 (ab150073, Abcam, Cambridge, UK). After three washes of 5 min each, VectaShield (H-1200, Vector Laboratories, Burlingame, CA, USA) were added to coverslips. Finally, coverslips were mounted and visualized in a LSM 800 microscope (Zeiss, Jena, Germany). 

### 2.12. Sonic Hedgehog Activation 

The next three groups were created to define the basal state of Sonic Hedgehog activation:

- Group I: negative control. It was transfected with a plasmid containing 8 times the mutated Gli1 promoter sequence (no Gli1 binding) followed by the firefly luciferase gene, the plasmid used as a loading control, renilla luciferase, and an empty control plasmid.
- Group II: experimental group. It was transfected with the plasmid containing 8 times the Gli1 wild-type promoter sequence followed by the firefly luciferase gene, the plasmid used as loading control, and an empty control plasmid.
- Group III: positive control. It was transfected with the plasmid that contained 8 times the sequence of the Gli1 wild-type promoter followed by the firefly luciferase gene, the plasmid used as loading control, and the Gli1-expression plasmid.

### 2.13. Autophagy by Western Blot

Cell lines were treated with 100 nM Bafilomycin A1 or DMSO as vehicle control for 6 h, and, after that time, proteins were extracted and run in a western blot. LC3B and β-actin expression was measured. 

The 72-h siRNA-treated GBM cell lines were then treated with 100 nM Bafilomycin A1, EBSS, EBSS + Bafilomycin A1, or DMSO as a vehicle control for 6 h. After that, proteins were extracted, and LC3B and β-actin was measured. 

### 2.14. Autophagy by Fluorescence

GBM cell lines were seeded in 12-well plates and were transfected the next day with 50 pmol siCTRL or siHDAC6 and 1 µg of EGFP-LC3B plasmid following lipofectamine 2000 protocol. At 48 h after transfection, cells were treated with 100 nM Bafilomycin A1, EBSS, EBSS + Bafilomycin A1, or DMSO as a vehicle control for 6 h, and autophagosome formation was measured in a EVOS microscope. Autophagosomes were counted using ImageJ software.

### 2.15. Statistical Analysis

Statistical analysis was performed with GraphPad Prism 8.0 Software. Studies that contained more than two groups were analyzed with a one-way analysis of variance (ANOVA) followed by Tukey’s post-hoc multiple comparison test. *p* < 0.05 was considered statistically significant. 

## 3. Results

### 3.1. HDAC6, Autophagic Markers, and Mesenchymal Markers Are Overexpressed in Glioblastoma Samples Compared with Normal Controls

#### 3.1.1. RT-qPCR

**Figure 1:**
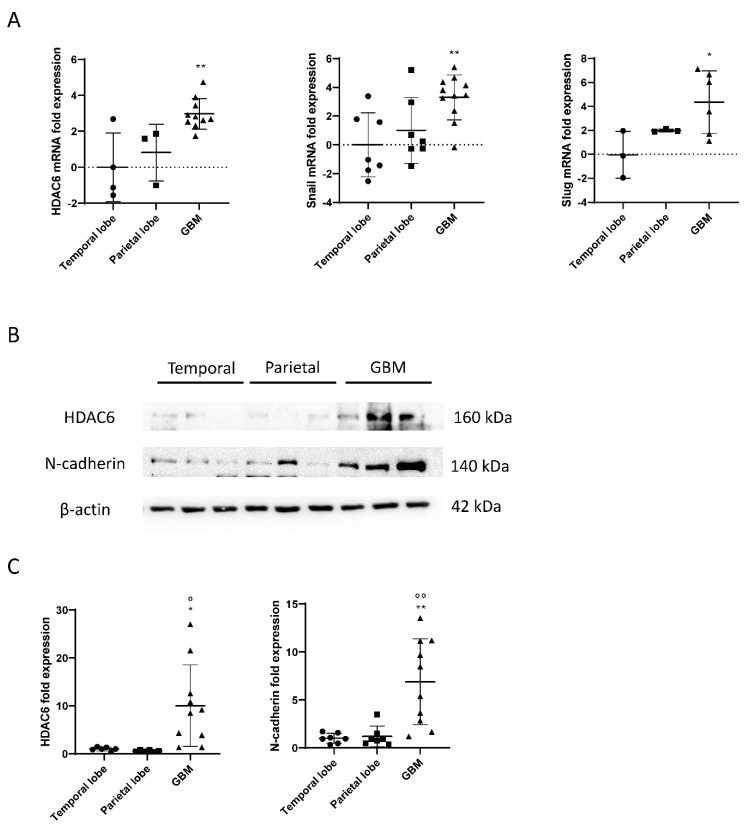
mRNA and protein overexpression of HDAC6 and mesenchymal markers in GBM samples. (**A**) RT-qPCR for HDAC6 and mesenchymal markers Snail and Slug in parietal and temporal lobe as well as GBM tissue. Data are represented as mean ± SD. (*) *p* < 0.05, (**) *p* < 0.01 vs. temporal lobe. (Temporal lobe: *n* = 7, parietal lobe: *n* = 7, GBM *n* = 11). (**B**) Western blot image for HDAC6, N-cadherin, and β-actin of control brain tissue and GBM tissue. (**C**) Analysis of western blot. Data are represented as mean ± SD. (*) *p* < 0.05, (**) *p* < 0.01 vs. temporal lobe, (⁰) *p* < 0.05, (⁰⁰) *p* < 0.01 vs. parietal lobe (temporal lobe: *n* = 7, parietal lobe: *n* = 7, GBM *n* = 10).

RNA was extracted from frozen samples of healthy brains and glioblastomas, and a reverse transcription (RT) and a real-time PCR (qPCR) were performed to study the expression of HDAC6 in those samples. As a result, we obtained that HDAC6 was three times more expressed in glioblastoma tissue than in tissues of the temporal area of healthy brains (*p* = 0.0038) (Figure 1). The differences when comparing with tissues of the parietal area of the brain were smaller, but the result obtained was not statistically significant (*p* = 0.053). It should be noted that we were unable to detect this expression in three of seven samples from the temporal area and in four of seven samples from the parietal area, which could have increased the differences and the statistical power.

**Figure 2:**
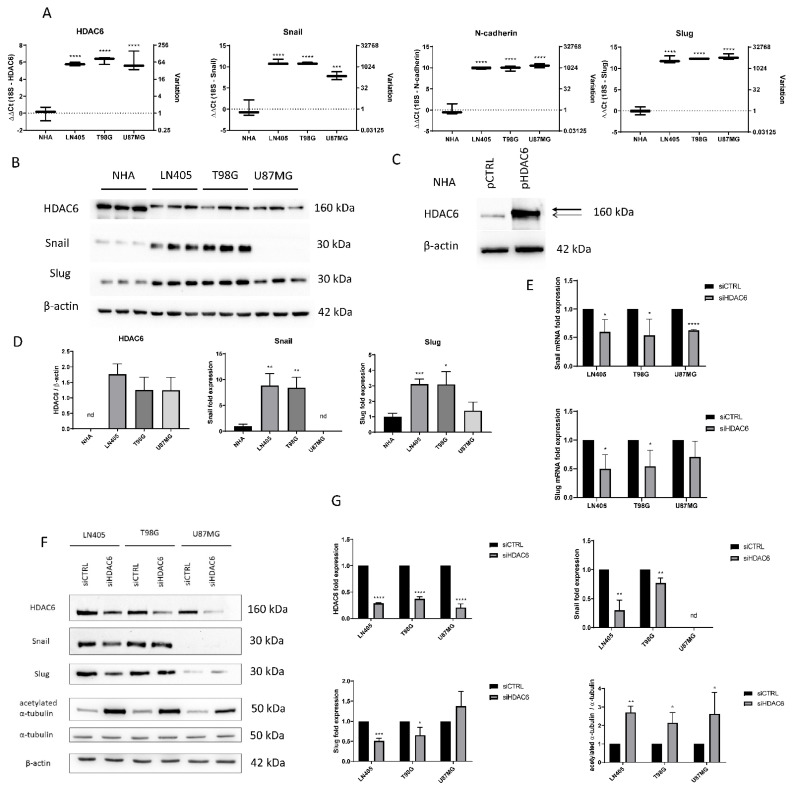
HDAC6 silencing decreases the overexpression of HDAC6 and mesenchymal markers in GBM cell lines. (**A**) Basal expression of mRNA levels of HDAC6, Snail, Slug, and N-cadherin in human GBM cell lines and NHA cell line. Data are represented as mean ± SD. (***) *p* < 0.001, (****) *p* < 0.0001 vs. NHA (*n* = 3). (**B**) Representative western blot image for HDAC6, Snail, Slug, and β-actin of human GBM cell lines and NHA cell line. (**C**) Western blot image of HDAC6 in control NHA- and pHDAC6-transfected NHA cell line. Thick line shows real HDAC6 band, while thin line shows the unspecific band. (**D**) Analysis of western blot. Data are represented as mean ± SD. (*) *p* < 0.05, (**) *p* < 0.01, (***) *p* < 0.001 vs. NHA. (*n* = 3). nd: not determined. (**E**) mRNA levels of Snail and Slug relativized to siCTRL after siRNAs treatment. (*) *p* < 0.05, (***) *p* < 0.001 vs. siCTRL condition. (**F**) Representative western blot image for HDAC6, Snail, Slug, acetylated α-tubulin, α-tubulin, and β-actin after treatment with siRNAs. (**G**) Analysis of western blot. Data are represented as mean ± SD. (*) *p* < 0.05, (**) *p* < 0.01, (***) *p* < 0.001, (****) *p* < 0.0001, vs. siCTRL condition (*n* = 3). nd: not determined.

Regarding the Snail and Slug genes, we obtained similar results as for the expression of HDAC6 (Figure 2). We observed that the Snail gene was about three times more expressed in glioblastoma tissue than in tissues of the temporal zone (*p* = 0.0062), and the differences between the parietal zone of the brain and glioblastoma were almost significant (*p* = 0.062). Slug was also found four times more expressed in the tumor tissue than in tissues of the temporal zone (*p* = 0.0426) and did not give a significant difference between tissues of the parietal zone and glioblastoma (*p* = 0.31).

#### 3.1.2. Western Blots

As explained, HDAC6 and the mesenchymal markers Snail and Slug were overexpressed in tumor tissue, as detected by RT-qPCR. To test the markers by western blot, proteins were extracted from healthy brain tissues and glioblastoma, and the expression of HDAC6 and N-Cadherin were quantified using β-actin as a loading control.

The results obtained by the RT-qPCR were correlated with the data obtained by western blot. Tumor tissues showed overexpression of HDAC6 in comparison with the healthy tissues of the temporal and parietal zones (Figure 1). Similar results were detected in the case of the mesenchymal marker N-Cadherin, since, despite not having been studied by RT-qPCR, its expression by western blot correlates with the overexpression of Snail and Slug markers by RT-qPCR.

#### 3.1.3. Immunohistochemistry

**Figure 3:**
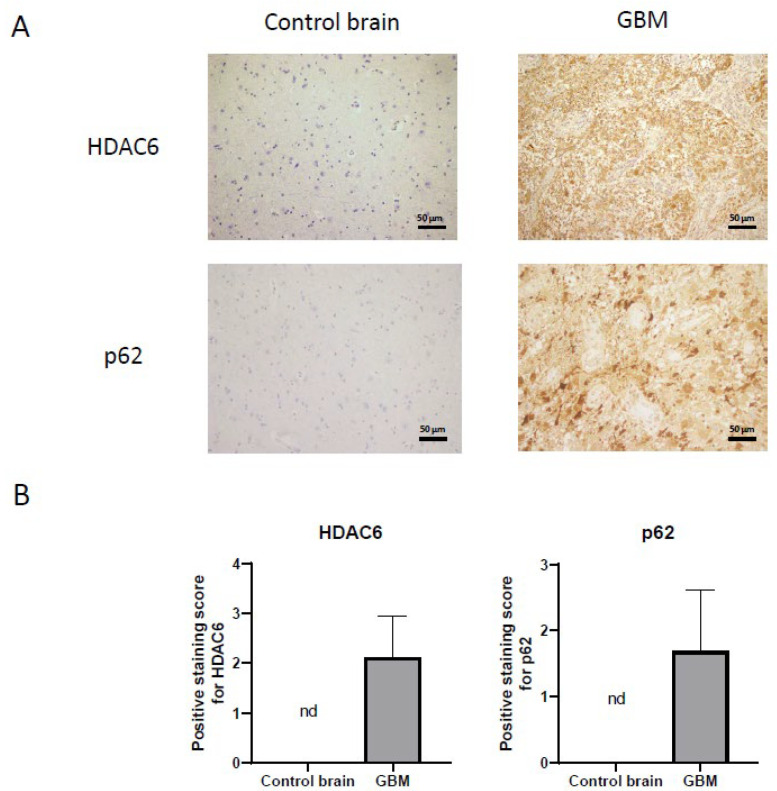
HDAC6 and autophagic marker p62 overexpression detected by immunohistochemistry in GBM samples. (**A**) Representative image of each protein and group. Every photograph has been taken at 20×. (**B**) Analysis of positive staining score of each sample. Data are represented as mean ± SD (control brain tissue: *n* = 10, GBM: *n* = 40). nd: not determined.

Once we demonstrated that HDAC6 is overexpressed at the RNA and protein level in GBM, we performed immunohistochemistry against HDAC6 in healthy brain sections and in GBM sections from the Hospital Complex of Navarra. As we see in Figure 3, healthy tissue did not present staining against this protein, while in tumor tissue, we did observe positive cytoplasmic staining and negative nuclear staining.

On the other hand, we also studied the autophagic marker p62 (Figure 3) by immunohistochemistry. For p62, as with HDAC6, we were unable to detect staining in healthy tissue, while we did see a very marked staining in tumor tissue, indicating that autophagy could be increased in this type of cancer. 

### 3.2. HDAC6, Autophagic Markers, and Mesenchymal markers Are Overexpressed in Glioblastoma Cell Lines Compared with a Normal Human Astrocytes Cell Line

#### 3.2.1. Baseline Status of Glioblastoma Cell Lines

In order to compare the basal state of HDAC6 expression and some mesenchymal markers among the cell lines used in our study, two types of independent experiments were carried out. First, an extraction of mRNA from the four cell lines and reverse transcription of it was performed. Once the cDNA was obtained, an RT-qPCR was made to quantify and compare the expression levels of HDAC6, Snail, Slug, and N-Cadherin genes. (Figure 2). HDAC6 levels were found to have higher expression in the three tumor cell lines compared to the normal human astrocytes (NHA) cell line. This result, like that obtained in the experiments with healthy and tumor tissues, confirms that this gene is overexpressed in glioblastoma. The mesenchymal markers Snail, Slug, and N-Cadherin also showed higher levels of expression in the tumor cell lines compared to NHA. No expression differences were seen between glioblastoma cell lines except in the Snail gene, which presented lower levels in the U87MG line.

For detection of expression at the protein level, the protein extract was quantified and the same amount of protein from each cell line was loaded on an SDS-PAGE to make a western blot. As in the RT-qPCR, we observed that the levels of Snail and Slug in LN405 and T98G cell lines were increased (Figure 2). Unexpectedly, we did not observe Snail expression in U87MG cells, while Slug expression in these cells showed similar levels as in NHA cells. This seems to indicate that LN405 and T98G cells have a mesenchymal phenotype, while U87MG cells have an epithelial phenotype.

Regarding the expression of HDAC6, at first glance, we witnessed a band in all cell lines and, surprisingly, this band was more intense in the non-tumor cells, which was not consistent with the data obtained both by RT-qPCR and western blot of the tissues. Interestingly, that same more intense band in the NHA line did not correspond to the height of HDAC6 but was slightly below the bands observed in the tumor cells (Figure 2). To check if this band was HDAC6, we transfected the NHA line with an HDAC6-expression plasmid or a control plasmid. Two days after transfection, proteins were extracted, and a western blot was performed to detect HDAC6 again. We observed that in the condition transfected with the HDAC6-expression plasmid, an overexpression of this protein occurred, and the band that appeared was just above the band presented by the group transfected with the control plasmid, which suggests that the latter band corresponds to non-specificity of the antibody and, therefore, does not correspond to HDAC6 (Figure 2). Concluding, we did not detect the presence of HDAC6 in NHA cells and, therefore, our glioblastoma cell lines certainly present HDAC6 overexpression.

#### 3.2.2. The U87MG Glioblastoma Cell Line Presents Sonic Hedgehog Pathway Activation

**Figure 4:**
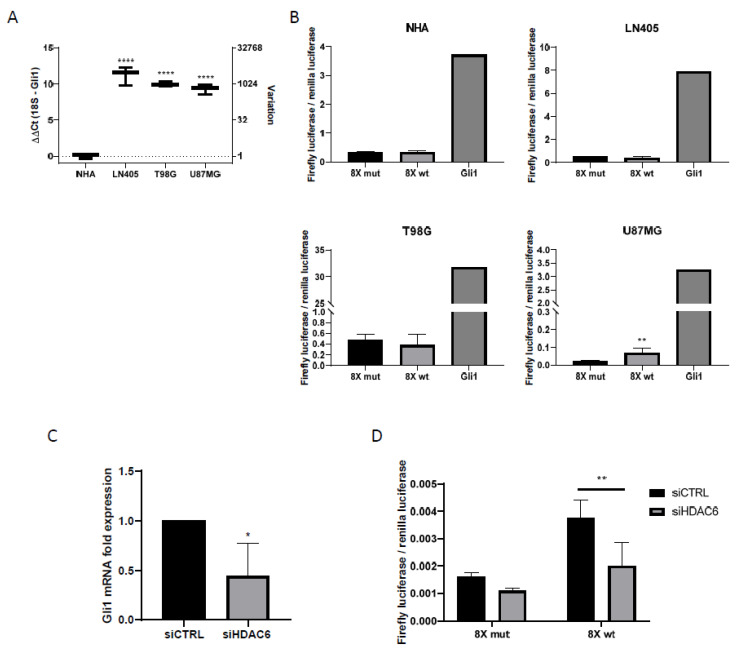
Shh pathway is only active in U87MG cells, and HDAC6 silencing inhibits this pathway. (**A**) RT-qPCR for Gli1 in human GBM cell lines and NHA cell line. Data are represented as mean ± SD (****) *p* < 0.0001 vs. NHA. (**B**) Gli1-luciferase assay in human GBM cell lines and NHA. Data are represented as mean ± SD (**) *p* < 0.01 vs. 8× mut (*n* = 4 of three independent experiments). (**C**) RT-qPCR for Gli1 in U87MG cell line after silencing of HDAC6. Data are represented as mean ± SD (*) *p* < 0.05 vs. siCTRL condition (*n* = 3). (**D**) Gli1-luciferase assay in U87MG cell line after treatment with siRNAs. Data are represented as mean ± SD (**) *p* < 0.01 (*n* = 4 of three independent experiments).

RT-qPCR showed Gli1 overexpression in glioblastoma cell lines (Figure 4). This led us to think that, since Gli1 was the common end target of the Sonic Hedgehog pathway, all three GBM-derived cell lines would have this pathway activated.

To check the basal state of the activation of the Sonic Hedgehog pathway, cells were transfected with various plasmids, creating three different groups detailed in Materials and Methods (negative control, positive control, and experimental group).

The day after transfection, the culture medium was changed to a medium that did not contain serum, to inhibit cell proliferation. The incubation time with serum-free medium was one day. After this time, the activities of the luciferases were measured with the Promega Dual-Glo luciferase kit (Promega, Madison, WI, USA). Of the four cell lines used in the experiment, only the U87MG cell line was shown to have the Sonic Hedgehog pathway activated, since it was the only cell line in which higher values of the firefly luciferase/renilla luciferase ratio were obtained in the group with the wild-type promoter versus the mutated one (Figure 4). In all cell lines, we observed how the highest value of luciferase activity corresponded to the group used as positive control, which shows the efficacy of the experiment.

#### 3.2.3. Glioblastoma Cell Lines Lack the Primary Cilium

Once we saw that the three tumor cell lines presented HDAC6 overexpression both at the mRNA and at the protein level, we also wanted to study if they had a well-formed primary cilium since HDAC6 has acetylated α-tubulin as its target, one of the structural proteins of the primary cilium. Therefore, we believe that the cilium is a target for HDAC6.

To do this, we seeded the cells in 24-well plates in which a coverslip previously sterilized with ethanol had been deposited. The following day, the culture medium was changed to new medium lacking serum and, after another 24 h, the coverslips were fixed with 4% formaldehyde. Immunofluorescence was performed against acetylated α-tubulin and γ-tubulin to visualize the primary cilium, using the NHA cell line as a positive control.

**Figure 5:**
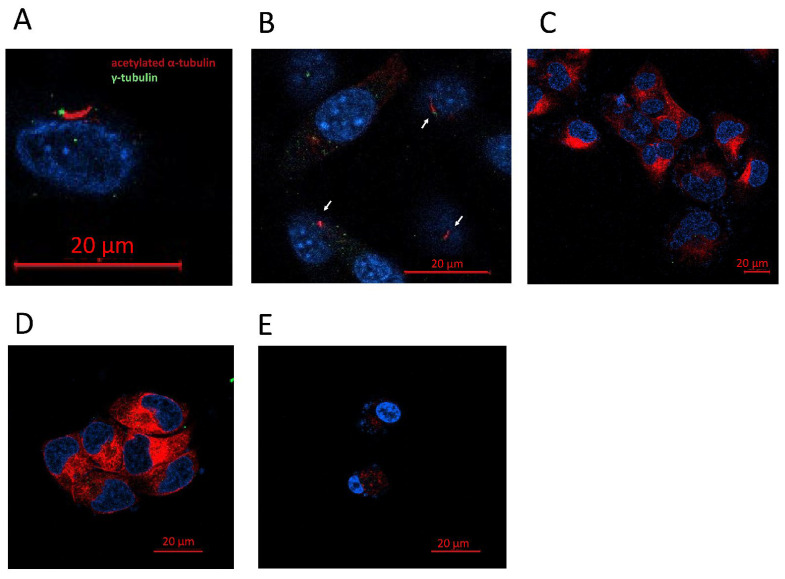
GBM cell lines lack a primary cilium. (**A**) Representative image of a primary cilium (NHA). (**B**) Presence of primary cilia in NHA cell line. (**C**–**E**) Human GBM cell lines did not present a primary cilium. (**C**) LN405, (**D**) T98G, and (**E**) U87MG.

The primary cilium can be identified if the basal body is detected: positive for γ-tubulin, close to the axoneme, and positive for acetylated α-tubulin as well (Figure 5). Of the four lines used in the study, we were only able to detect the presence of the primary cilium in the NHA cell line (Figure 5). The tumor cell lines LN405, T98G, and U87MG did not present a primary cilium, indicating that the loss of this structure might be oncogenic.

#### 3.2.4. Glioblastoma Cell Lines Show a High Level of Autophagy

As we have previously seen in immunohistochemistry in healthy brain and glioblastoma sections, the autophagy marker p62 is overexpressed in tumor tissue (Figure 3). This led us to think that the autophagy process would be increased in the tumor compared to healthy tissue.

To study this process, we created two experimental groups for each cell line. The first group was the control group without treatment. The second group was treated with 100 nM bafilomycin A1, a V-ATPase inhibitor, which prevents acidification of the autophagosome and its subsequent degradation, thus inhibiting the autophagic process. We used LC3B as autophagy marker in its lipidated variant, LC3BII, since it is the one found in the phagosome membrane.

The reason why we decided to carry out these experimental groups was the dynamism of autophagy. Autophagosomes are in a constant process of creation and destruction. Therefore, if we block autophagy, we will see an accumulation of autophagosomes in the cell. This difference in autophagosomes between the control and the treated group would be equivalent to autophagic flux; that is, autophagosomes that should have been degraded in baseline conditions were not finally degraded due to the inhibitory action of the drug. Therefore, the greater the difference in the levels of LC3BII between the control group and the group treated with bafilomycin A1, the greater autophagic activity the cell line will present.

**Figure 6:**
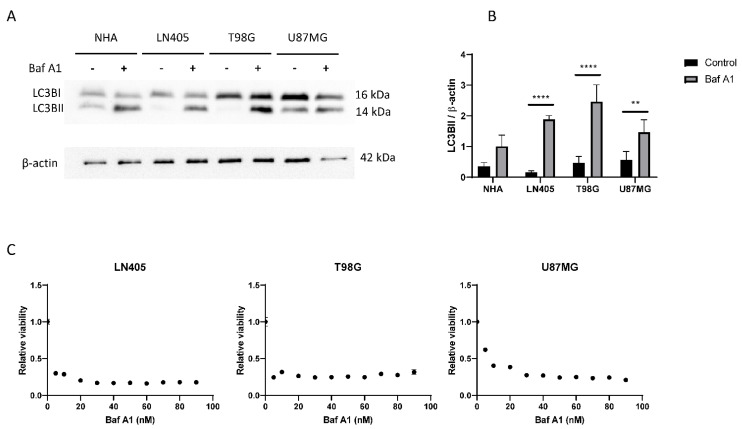
The autophagic flux is increased in GBM cell lines. (**A**) Representative image of western blot for LC3B and β-actin of human GBM cell lines and NHA cell lines after 6 h of 100 nM Baf A1 treatment. (**B**) Analysis of LC3BII western blot. Data are represented as mean ± SD (**) *p* < 0.01, (****) *p* < 0.0001 (*n* = 3). (**C**) Analysis of viability in human GBM cell lines after Baf A1 treatment for 72 h. Data are represented as mean ± SD (*n* = 3 of three independent experiments).

Consistent with what was observed in the immunohistochemistry of autophagic markers in tissues, increased autophagy was observed in tumor lines LN405, T98G, and U87MG (Figure 6), a result that makes us think that autophagy might be an oncogenic process.

Once we saw that the autophagic process was increased in tumor cell lines, we decided to study whether its blockade inhibits cell proliferation. To do this, we seeded cells from the three cell lines in 96-well plates, and the next day, they were treated with increasing concentrations of bafilomycin A1. Three days after treatment, an MTT was performed to visualize cell viability. We saw that all cell lines suffered a great decrease in cell viability in those conditions treated with bafilomycin A1 (Figure 6), which shows that the autophagic process is necessary for proliferation in these three tumor cell lines.

### 3.3. Effect of HDAC6 siRNA Silencing in Glioblastoma Cell Lines

#### 3.3.1. siHDAC6 Inhibits HDAC6 Expression and Activity in Glioblastoma Cell Lines

After observing an increase in the expression of HDAC6 both at the mRNA level and at the protein level (Figure 2) in tumor tissues versus healthy tissues and in the cell lines derived from glioblastoma versus the cell line of normal astrocytes (NHA), we set out to modulate their expression through the use of siRNA to see the effect that silencing could have on the oncogenic capacity of glioblastoma tumor lines.

For this purpose, cells of the three tumor cell lines were seeded in 6-well plates. The next day, they were silenced with a mixture of two siRNAs that targeted HDAC6 mRNA (siHDAC6) or with a control siRNA (siCTRL) that has no affinity for any mRNA sequence. Silencing was done using the lipofectamine 2000 method. Four hours after incubation with the siRNAs, the medium was changed and allowed to incubate for 72 h. After this time, the cells were subjected to different experiments.

RNA was extracted from the silenced cells with the control siRNA or with the mixture of HDAC6 siRNAs. This RNA was subjected to RT-qPCR to analyze the expression status of HDAC6 after silencing in the three cell lines. We observed that in the three cell lines there was a decrease in the expression of HDAC6 (between 50% and 80%) in the cells treated with siHDAC6 compared with those silenced with siCTRL (Figure 2).

Once we saw that the silencing with siHDAC6 significantly decreased the expression of the HDAC6 mRNA, we wanted to see if this translated into a decrease in the protein level. For this, 72 h after treatment with siRNAs, protein extraction and a western blot were performed. This experiment confirmed the data obtained by means of RT-qPCR, since there was a decrease in the protein level of HDAC6 levels in the condition treated with siHDAC6 (Figure 2). Furthermore, this decrease in HDAC6 levels led to an increase in the ratio of acetylated α-tubulin/total α-tubulin, since acetylated α-tubulin is a target of HDAC6. Therefore, we can conclude that silencing by siHDAC6 has significant effects on its expression at both the protein and mRNA levels and on its activity.

#### 3.3.2. siHDAC6 Decreases Cell Proliferation and Clonogenicity in Glioblastoma Cell Lines

After demonstrating that treatment with siHDAC6 decreases the expression of this protein, we studied the effect of silencing on proliferation and the ability to form colonies in tumor lines. To do this, we seeded the cells, and the next day, we performed the silencing with siRNAs of HDAC6 or the control. At 72 h after the treatment, we trypsinized the cells, counted, and replated them both in 96-well plates to measure viability and in 6-well plates to perform a colony formation assay.

**Figure 7:**
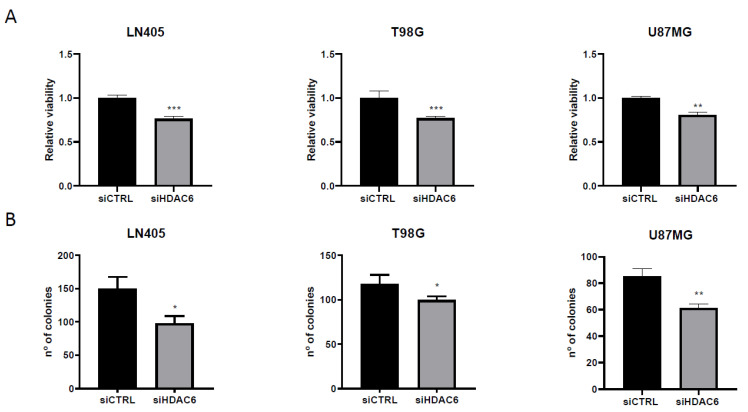
HDAC6 silencing reduces viability and clonogenic capacity in GBM cell lines. (**A**) Analysis of viability after 72 h of siRNAs treatment. (**) *p* < 0.01, (***) *p* < 0.001 vs. siCTRL condition (*n* = 4 of three independent experiments). (**B**) Analysis of number of colonies counted after siRNAs treatment. Data are represented as mean ± SD (*) *p* < 0.05, (**) *p* < 0.01 vs. siCTRL condition (*n* = 3 of three independent experiments).

For the proliferation assay, one day after plating cells, an MTT assay was performed to define the viability value at time zero and thus have a reference value. The MTT test was done on another plate 72 h after measuring the time zero plate. The value of time zero was subtracted from each value, and it was relativized to the results of the control group. As a result, we obtained that the silencing of HDAC6 inhibits cell growth (Figure 7), which makes us think that, by promoting proliferation, HDAC6 has an oncogenic role and therefore may be a therapeutic target in glioblastoma.

On the other hand, seeing the oncogenic potential that HDAC6 has, we hypothesized whether, by silencing this protein, we could decrease the oncogenic activity of cell lines and decrease their clonogenic capacity. To do this, after seeding the silenced cells in the 6-well plates, they were incubated for 10 days so that they grew forming colonies. After this time, they were fixed with 4% formaldehyde and stained with crystal violet. The next day, the colonies formed during this time were counted. We saw that, as in the case of proliferation, the condition treated with siHDAC6 presented fewer colonies than the control condition (Figure 7). These results indicate that HDAC6 is a protein involved in cell growth and in the oncogenic capacity of these cell lines derived from glioblastoma.

#### 3.3.3. siHDAC6 Decreases Cell Migration in Glioblastoma Cell Lines

**Figure 8:**
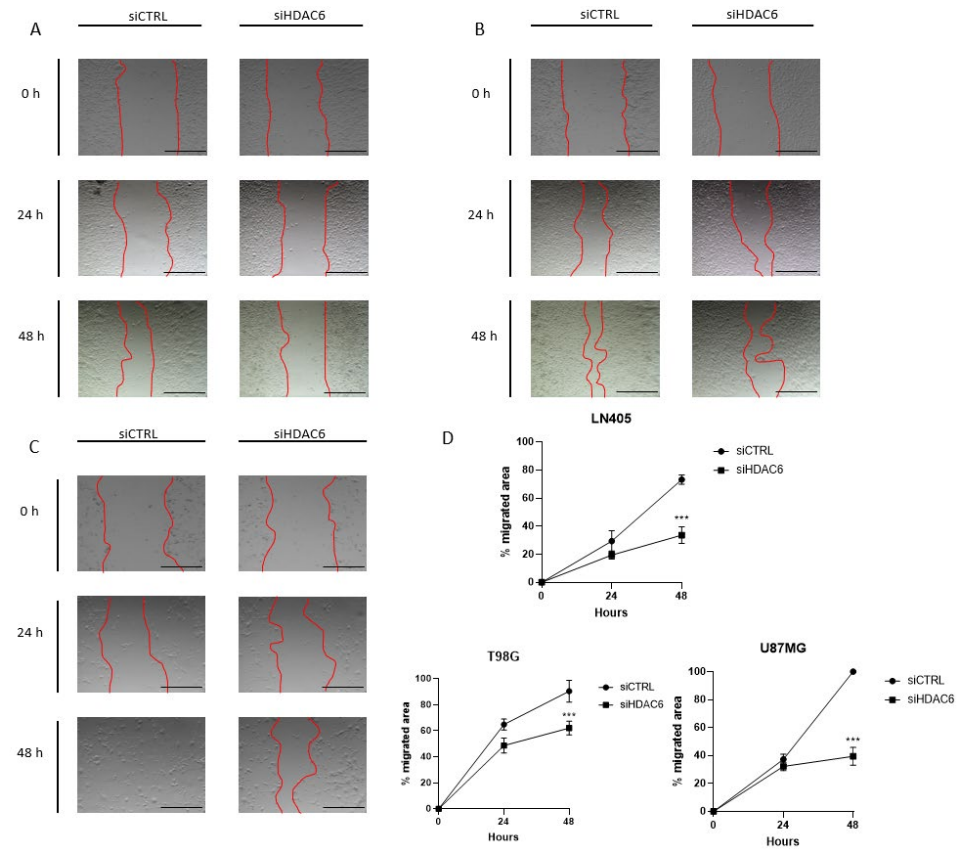
HDAC6 silencing inhibits the migration potential of GBM cell lines. (**A**–**C**) Representative images of wound healing assay at 0, 24, and 48 h post-scratch. The red bars represent the migration front in (**A**) LN405, (**B**) T98G, and (**C**) U87MG. (**D**) Analysis of wound closure assay. Scale bars drawn on the pictures correspond to 500 µm. Data are represented as mean ± SD. (***) *p* < 0.001 vs. siCTRL condition at same time (*n* = 3 of three independent experiments).

Once we saw that the silencing of HDAC6 by interfering siRNAs decreases cell proliferation and the clonogenic capacity of tumor cell lines, we wondered if this silencing could also decrease cell migration. To study this effect, the same procedure was followed in the silencing as in the study of HDAC6 expression and of proliferation and clonogenic capacity after treatment with siCTRL or siHDAC6. In this case, the cells were trypsinized, counted, and seeded in 24-well plates until fully confluent. The next day, the monolayer was wounded and the culture medium was changed to another with 2.5% FBS to inhibit proliferation. When the wound was made, a photograph of the well was taken at time zero; at 24 and 48 h after the wound was made, photographs were taken again. HDAC6 silencing decreased the migratory capacity of the cells after wounding (Figure 8). This difference was seen in the three cell lines after 48 h, although it was noticeable in the U87MG line 24 h after the wound was made. With these results, we can affirm that HDAC6 promotes cell migration, and its silencing reduces the pro-migratory effect.

#### 3.3.4. siHDAC6 Reverses the Epithelial-to-Mesenchymal Transition in Glioblastoma Cell Lines

We have previously observed that the levels of some mesenchymal markers, such as N-cadherin, Snail, and Slug, are overexpressed in glioblastoma samples and tumor cell lines both at the mRNA level and at the protein level. We then wondered if we could inhibit its expression and thus revert the mesenchymal phenotype after HDAC6 silencing by siRNA.

To do this, we made an RT-qPCR for Snail and Slug genes in the three cell lines, comparing their expression between the control group and the group in which we silenced HDAC6. We observed a decrease in Snail in all three tumor lines. In the case of Slug, the decrease was also observed but only in LN405 and T98G. The U87MG line did not show significant differences in the expression of this marker between the siCTRL and siHDAC6 groups (Figure 2).

Once we saw the decrease in these markers at the mRNA level, we made the same comparison at the protein level to see if we also observed this decrease after HDAC6 silencing. We used the protein extracts used to study the protein expression of HDAC6 after treatment with the different siRNAs, and we performed a western blot against the same proteins as in the comparison by means of RT-qPCR. We observed that the Snail and Slug markers decreased in the LN405 and T98G cell lines, which are the ones with a more marked mesenchymal phenotype. In the case of U87MG, we did not detect a decrease in the Slug protein, while, as in the comparison between the cell lines in the basal state, we were not able to detect Snail (Figure 2E–G). These results seem to indicate that HDAC6 promotes the epithelial-to-mesenchymal transition, and that, after HDAC6 silencing, we are able to reverse this process.

#### 3.3.5. siHDAC6 Restores the Primary Cilium in Glioblastoma Cell Lines

**Figure 9:**
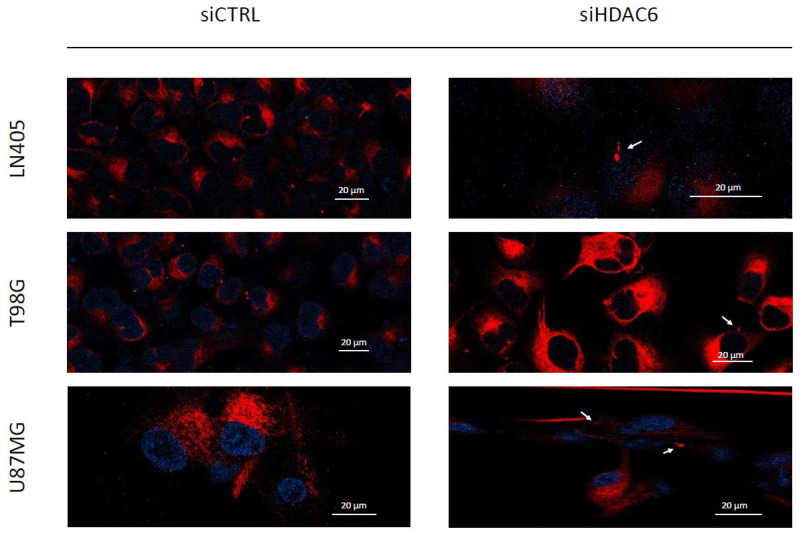
HDAC6 silencing restores a primary cilium-like structure in GBM cell lines. The white arrows mark the primary cilium-like structure in siHDAC6-treated human GBM cell lines. Photographs were taken at 63×.

Primary cilia are present in normal human astrocytes, but we have not been able to detect them in glioblastoma cells. Acetylated α-tubulin is one of the structural proteins of the primary cilium and is deacetylated by HDAC6. As we have seen in the comparison between cell lines and between healthy and tumor brain tissues, HDAC6 is overexpressed in glioblastoma, and this overexpression could lead to a decrease in acetylated α-tubulin levels and, consequently, to the absence of primary cilium in glioblastoma cell lines. Therefore, we believe that if we silence HDAC6, we could be able to restore the presence of the primary cilium in the glioblastoma cell lines. In order to test our hypothesis, we seeded cells in 24-well plates in which we had previously deposited a coverslip sterilized with ethanol. The next day, we did the silencing by dealing with siCTRL or the siHDAC6 mix. Two days after silencing, we changed the medium to serum-free medium to induce ciliogenesis and allowed to incubate for an additional day. After this time, we fixed the cells with 4% formaldehyde and performed immunohistochemistry against acetylated α-tubulin and γ-tubulin. We saw that none of the cell lines treated with siCTRL had cilium, corroborating what was seen in the comparison of the non-tumor cell line (NHA) with the glioblastoma cell lines. However, after HDAC6 silencing, a small subpopulation of cells presented a structure similar to the primary cilium, although we could not observe it in most of the cells (Figure 9). With these results, we might conclude that HDAC6 is an important protein in the process of aberrant ciliogenesis, and it seems to be involved in the disappearance of the primary cilium, but that is not the only determining factor in this process.

#### 3.3.6. siHDAC6 Decreases the Activity of the Sonic Hedgehog Pathway in Glioblastoma Cell Lines

As seen before, U87MG is the only one of the three cell lines in the study that exhibited activation of the Sonic Hedgehog pathway. To see if there was a relationship between the Sonic Hedgehog pathway and HDAC6 expression, we decided to study the expression of Gli1 by RT-qPCR in samples treated with siCTRL and with the mixture of siHDAC6 in U87MG cells.

Gli1 expression decreased significantly when silencing HDAC6 compared to the control group (Figure 4), which seemed to indicate that there was a decrease in the activity of the pathway. In order to verify this, we seeded the U87MG cell line and transfected it with the plasmids of the wild-type and mutated Gli1 promoters followed by the firefly luciferase gene, the renilla luciferase plasmid, and the siRNAs of the study, creating four experimental groups. Two groups contained the mutated Gli1 promoter plasmid and the renilla luciferase plasmid and each of the groups was also transfected with either siCTRL or siHDAC6. The same groups were created by exchanging the mutated Gli1 promoter for the wild-type promoter.

In this case, we also saw a decrease in the quotient of firefly luciferase and renilla luciferase in the group in which we had silenced HDAC6 compared to the control group (Figure 4), which confirms the result obtained by RT-qPCR. Also, as in the case of the comparison of the Sonic Hedgehog pathway between cell lines (Figure 4), the control condition that carried the Gli1 wild-type promoter presented greater activity than the control condition that contained the promoter of mutated Gli1, reconfirming the activation of the pathway in the U87MG line. With these results, we can conclude that HDAC6 promotes the activation of the Sonic Hedgehog pathway, and its silencing decreases the activity of this signaling pathway.

#### 3.3.7. siHDAC6 Blocks Autophagy in Glioblastoma Cell Lines

**Figure 10:**
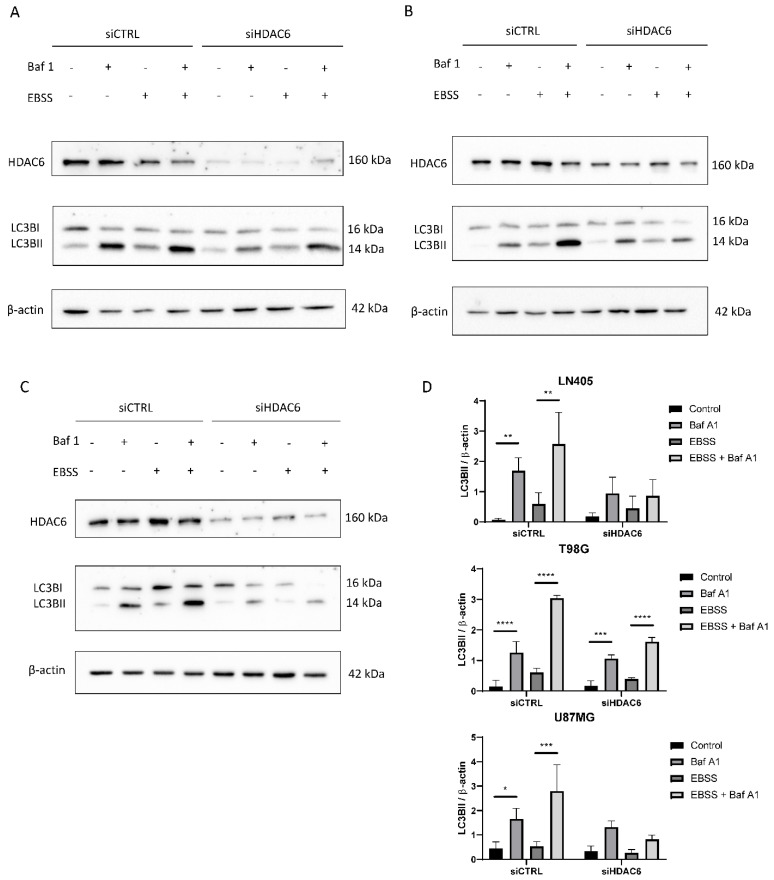
HDAC6 silencing decreases the autophagic flux in GBM cell lines. (**A**–**C**) Representative image of western blot of HDAC6, LC3B, and β-actin after 100 nM Baf A1, EBSS, EBSS + Baf A1, or DMSO (as vehicle control treatment) for 6 h in (**A)** LN405, (**B**) T98G, and (**C**) U87MG cell lines. (**D**) Analysis of LC3BII western blot. Data are represented as mean ± SD (*) *p* < 0.05, (**) *p* < 0.01, (***) *p* < 0.001, (****) *p* < 0.0001 (*n* = 3).

Immunohistochemical analysis demonstrated that autophagic markers are overexpressed in glioblastoma samples compared to healthy brain tissue (Figure 3). Furthermore, in the comparison between the cell lines, we also saw that autophagy is increased in the three cell lines compared with the non-tumor cell line (NHA) (Figure 10). Therefore, we wonder if HDAC6 plays an important role in the induction of autophagy.

In order to answer our question, we reused the RNA samples extracted after silencing with siCTRL and siHDAC6 and performed an RT-qPCR to see if p62 expression had changed. In those samples in which we silenced HDAC6, p62 expression decreased. In conclusion, HDAC6 might be related to autophagy.

Once we saw that HDAC6 had an effect on the expression of the marker p62 at the mRNA level, we tried to see if we really observed differences at the protein level after inhibition and stimulation of autophagy while silencing HDAC6. To do this, we seeded the cells and the next day, we performed the silencing with siCTRL or with the mixture of siHDAC6. Three days after silencing, different treatments were performed to observe autophagy in the different groups (siCTRL and siHDAC6). The first treatment was the control, which only carried DMSO; the second group was treated with bafilomycin A1 at a concentration of 100 nM; the third group was incubated with a nutrient-deficient medium, EBSS, to stimulate the autophagic process; and the last group was treated with 100 nM bafilomycin A1 in the EBSS nutrient-deficient medium. These treatments lasted 6 h. After this time, a protein extraction was carried out and was quantified the following day. With these protein extracts, a western blot was performed against the LC3B marker.

In the LN405 cell line, a very low expression of the marker LC3BII was observed in basal conditions, which was increased when it was treated with the inhibitor bafilomycin A1 (Figure 10). This is because the inhibitor does not allow the degradation of autophagosomes, and that is why we see the accumulation of LC3BII. In the case of EBSS treatment, the expression of LC3BII was increased with respect to the control group. We had the greatest presence of LC3BII in the EBSS + bafilomycin A1 treatment, which was expected because in this condition we stimulated autophagy, since there are no nutrients and the cell needs to obtain energy; on the other hand, we are not allowing those autophagosomes to degrade. In the case of the silencing of HDAC6, we see that in the conditions in which we treated with bafilomycin A1, there is an increase in LC3BII, but it is not as noticeable as in the same condition silenced with siCTRL.

In the case of the T98G cell line, we obtained similar results to those obtained in LN405 cells. The expression of LC3BII increased in all the conditions in which we had carried out the treatment with bafilomycin A1, and the highest expression was found in the silenced condition with siCTRL and treated with EBSS + bafilomycin A1 (Figure 10). Although, unlike the LN405 line, only a slight decrease in autophagy was observed after HDAC6 silencing compared to the control group, a large decrease in the condition of the EBSS + bafilomycin A1 treatment was observed in the cells treated with the mixture of siHDAC6 compared to the control group.

The U87MG cell line also showed a decrease in autophagy when HDAC6 was silenced. Again, the difference in expression of LC3BII between the control and bafilomycin A1 groups was slightly lower in the conditions treated with the mixture of siHDAC6, while this difference was more noticeable between the EBSS and EBSS + bafilomycin A1 conditions (Figure 10). We can thus affirm that HDAC6 is a protein that is positively involved in the autophagy process. When we silence its expression, we manage to partially block the autophagic process.

To give greater validity to the results, we decided to make an observation of the formation of autophagosomes by means of a fluorescence experiment. To do this, we seeded the cells, and the next day we co-transfected them with a plasmid that expressed the EGFP-LC3B fusion protein and with each of the siRNAs of the study, siCTRL, or siHDAC6. In this case, the period after the transfection in which we obtained the images was 48 h, since at 72 h, we observed great cell death. At 48 h after transfection, we performed the treatments with bafilomycin A1, EBSS, EBSS + bafilomycin A1, and DMSO as a control group. After 6 h incubation with the treatment, the autophagosomes were visualized in an EVOS microscope, and photographs of four random planes per well were taken, counting the amount of autophagosomes per cell in each of the conditions.

**Figure 11:**
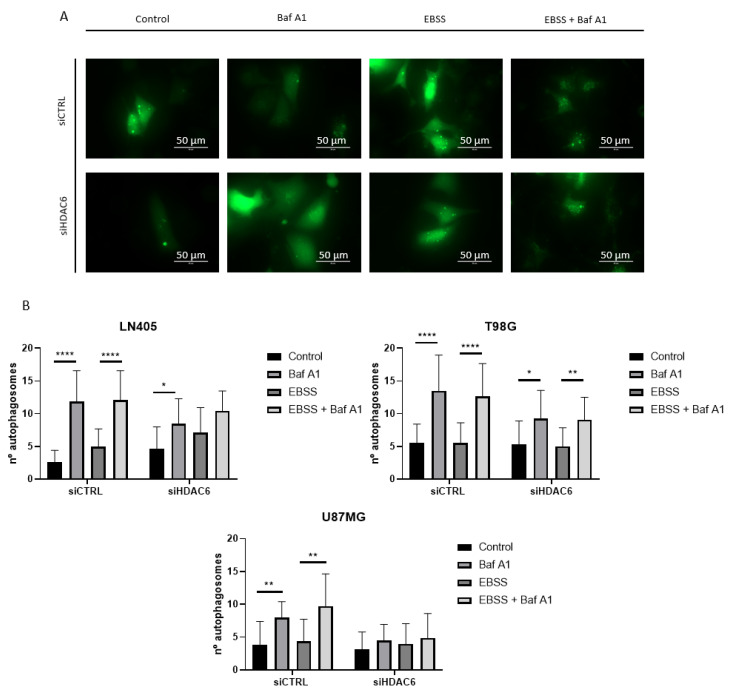
HDAC6 silencing decreases the number of autophagosomes per cell. (**A**) Representative image of a cell with autophagosomes after siRNAs treatment for 72 h and 100 nM Baf A1, EBSS, EBSS + Baf A1, or DMSO (as vehicle control) treatment for 6 h in LN405. Photographs were taken at 60×. (**B**) Analysis of the number of autophagosomes per cell. Data are represented as mean ± SD (*) *p* < 0.05, (**) *p* < 0.01, (****) *p* < 0.0001.

The results we obtained corroborated the data obtained by western blot. In those conditions in which HDAC6 was silenced, the blockage of autophagy due to bafilomycin A1 treatment induced a lower number of autophagosomes per cell (Figure 11 and Figure S1). This confirms that HDAC6 silencing decreases autophagy in these glioblastoma cell lines, although we do not know if this is due to the fact that silencing inhibits autophagosome formation or inhibits the onset of autophagy. For solving this question, we would need to carry out further studies.

## 4. Discussion

### 4.1. Expression of HDAC6 and Mesenchymal and Autophagic Markers in Glioblastoma Samples and Cell Lines

Wang et al. [26] observed that HDAC6 was overexpressed in glioblastomas compared to healthy brain tissue, and, in addition, it was also overexpressed at the mRNA level in an online database search. Another study carried out in tissues of healthy and tumor controls demonstrated that HDAC6 appeared to be the isoform of HDAC most highly overexpressed in glioblastoma [3]. To verify this fact, we performed immunohistochemistry against HDAC6 in paraffin sections of healthy brain tissue and of tumor tissue, and we detected overexpression of HDAC6 in glioblastoma, showing a cytoplasmic staining, which is consistent with the literature studied. To corroborate this fact, we studied the expression of HDAC6 both at the mRNA level and at the protein level, obtaining a similar result and confirming the overexpression of HDAC6 in glioblastoma. 

At the immunohistochemical level, we also studied the expression of different autophagic markers, such as p62 or LC3B. Autophagy is a regulatory mechanism usually increased in cancer cells to obtain the large amounts of energy needed for tumor development. An increase in autophagic markers in tumor tissue compared to healthy brain tissues is expected. This was the case with our immunohistochemical results of p62, a protein that showed overexpression in tumor tissue and is related to an advanced stage of the disease and to a worse prognosis in gliomas [27]. However, this did not occur for LC3B, as this antibody stains both the cytoplasmic form (LC3B-I) and the lipidated form found in the membrane of autophagosomes (LC3B-II). For this reason, in immunohistochemistry, we cannot differentiate between the two forms of LC3B and, therefore, whether or not there is an increase in autophagy. Even so, with these data, we believe that autophagy is a process that is increased in glioblastoma, and, therefore, can help tumor development and growth.

Thirdly, we also carried out a study of mesenchymal markers at the mRNA and protein levels. EMT is a reversible process that changes the cellular phenotype to a more undifferentiated phenotype and is usually accompanied by increased migration. In this case, using RT-qPCR, we studied the expression of the EMT-TFs Snail and Slug, seeing they were increased in glioblastoma. This overexpression has also been detected in other tumor types, such as breast [28], thyroid [29], esophagus [30], and colon [31] cancer, among others. At the protein level, we were able to confirm again the overexpression of the mesenchymal marker N-cadherin in glioblastoma. N-cadherin is also overexpressed in advanced stages of prostate [32], colorectal [33], and ovarian [34] cancer and in the latter after stimulation of the EGFR pathway, a protein that is overexpressed in glioblastoma and could be one of the causes of the EMT phenotype in this tumor. Thus, we can confirm that EMT is an active process in glioblastoma, and its inhibition could be beneficial for delaying tumor development and sensitizing glioblastoma to chemotherapy [35].

After having detected the overexpression of HDAC6 and mesenchymal and autophagic markers in tumor samples, we studied the expression levels of HDAC6 and of the mesenchymal markers Snail and Slug in our glioblastoma cell lines by RT-qPCR and western blot. Confirming the data obtained in tissue samples, we observed that both HDAC6 and mesenchymal markers were overexpressed in cell lines derived from glioblastoma. This result is consistent with previous studies conducted on other glioblastoma cell lines [26,36]. Interestingly, U87MG cells presented lower values of mesenchymal markers, and we did not even detect Snail expression at the protein level. This indicates that U87MG cells might be more epithelial in phenotype than LN405 and T98G cells.

The primary cilium, whose loss is associated with the dysregulation of several signaling pathways, such as the Shh pathway, is a structure responsible for capturing signals from the outside of the cell and for regulating the cell cycle. To verify the presence of the primary cilium in our four cell lines, we performed immunofluorescence against acetylated α-tubulin, a structural protein of the cilium. Only NHA cells presented a well-formed primary cilium. The three glioblastoma cell lines lacked a primary cilium. These data agree with previous studies carried out in patient tissues and in glioblastoma-derived cell lines [6,7,37]: despite the fact that some ciliated cells were detected in tumor tissues, we were not able to detect any in our glioblastoma cell lines, which leads us to think that the primary cilium acts as a tumor suppressor in glioblastoma. Furthermore, we believe that the absence of the primary cilium may be linked to the overexpression of HDAC6, since this enzyme has acetylated α-tubulin, a structural protein of the primary cilium, as its substrate. Absence of the primary cilium has also been reported in other tumor types, such as cancer of the pancreas [38], ovary [14], lung [12], kidney [39], and melanoma [40]. In addition, these tumors present overexpression of HDAC6 [40,41,42,43,44], like glioblastoma, which reinforces the hypothesis that the primary cilium disappears due to overexpression of HDAC6.

The Shh pathway is involved in several cellular processes, such as embryonic development, cell division, and cancer [45,46]. To check if the Shh pathway is active in our tumor cell lines, we performed an RT-qPCR against Gli1 and a luciferase assay. Although we detected overexpression of Gli1 in the three tumor cell lines, by means of the luciferase assay, we observed that this pathway was only active in the U87MG line. This result contrasts with the fact that the Shh pathway promotes EMT [47], as it is precisely the U87MG line, the most epithelial of the three glioblastoma cell lines, that presents an active Shh pathway. However, T98G cells have been shown to be activated with recombinant Shh treatment but not inhibited after GANT61 treatment [48], which we believe reinforces our results.

The baseline state of autophagy was the last comparison we made between the NHA and the glioblastoma cell lines. Autophagy is generally used by tumor cells to obtain the energy necessary to maintain the high rate of proliferation. We observed that in the three cell lines derived from glioblastoma, this process was more active than in the normal cell line. This corroborates our previous results obtained by immunohistochemistry, in which we saw that an autophagic marker, p62, presented a greater staining in tumor tissue than in healthy tissue. However, these results contrast with the data obtained by Huang et al., in which they show that as the grade of the astrocytic tumor increases, autophagy decreases [49]; but they agree with the data offered by Tamrakar et al., which indicate an increase in autophagic markers in high-grade gliomas compared to low-grade gliomas [50]. The conclusion reached by Huang et al. [49] is due to their observation of a decrease in beclin1 and LC3BII as tumor grade increased. We believe that these data are insufficient to be able to conclude that glioblastoma reduces autophagy since autophagy is a dynamic process in which autophagosomes, the storing bodies of LC3BII, are continuously being formed and degraded. Thus, one might observe a decrease in LC3BII due to the rate at which autophagosomes are degraded, but if their degradation were blocked, more autophagosomes could be stored at the same time, indicating a greater flow of autophagy. Interestingly, in our results, we barely detected LC3BII in the LN405 and T98G cell lines under baseline conditions, but after inhibition of autophagy, its expression increased even more than in the NHA cells, which, initially, had more LC3BII. Thus, by comparing the differences in LC3BII expression between the control condition and the condition treated with bafilomycin A1, we can confirm whether a cell line exhibits greater autophagy activity or not.

With these data, we have been able to determine that the glioblastoma cell lines used in our study exhibit HDAC6 overexpression, which we believe leads to defective ciliogenesis. Furthermore, although the three tumor cell lines express the mesenchymal markers Snail and Slug at the mRNA level, only U87MG cells lack an increase in their expression at the protein level, presenting a more epithelial phenotype than LN405 and T98G cells do. Of these three cell lines, only U87MG shows activation of the Shh pathway in the basal state despite being the most epithelial one. Furthermore, the glioblastoma-derived cell lines show overexpression of the autophagic process compared to the NHA cells. 

### 4.2. HDAC6 Silencing in Glioblastoma Cell Lines

After having verified that HDAC6 is overexpressed in glioblastoma, we wanted to silence its expression using a siRNA to infer the effects this protein might contribute to the development of this tumor. After using the control siRNA or the mixture of HDAC6 siRNAs, we measured the expression of HDAC6 both at the mRNA and protein levels, and we observed that the HDAC6 siRNA mixture was effective in reducing the expression of this protein. Decreased HDAC6 expression was associated with increased expression of acetylated α-tubulin, since this is a target of HDAC6. With this experiment, we observed that our silencing system not only decreased the expression of HDAC6 but could also decrease its activity. Soon after, we carried out several experiments to study the effect of HDAC silencing on different processes that promote the development of glioblastoma.

One of the effects of HDAC6 silencing in glioblastoma is the inhibition of cell proliferation and, therefore, the inhibition of tumor growth. These results have been observed in different tumor types, such as cholangiocarcinoma [51], lung cancer [52], and colon cancer [53]. Furthermore, the inhibition of HDAC6 not only decreased the proliferation of tumor cell lines but also their clonogenic capacity, demonstrating its oncogenic potential in glioblastoma.

Also, silencing of HDAC6 decreased cell migration, as has already been demonstrated in colon cancer [54,55] after treatment with a HDAC6 inhibitor, A452. A similar study was carried out in kidney cancer, showing an increase of cell migration after HDAC6 overexpression [56]. The decrease in acetylated α-tubulin levels is associated with greater cell migration in breast cancer [57,58]. In line with these results, we have seen that after silencing HDAC6 and, consequently, after increasing the levels of acetylated α-tubulin, we succeeded in inhibiting glioblastoma cell migration. 

HDAC6 is necessary for the induction of EMT after treatment with TGFβ in lung cancer [59] and in a non-tumor cell line [60]. Our HDAC6-silencing experiments induced a decrease in Snail and Slug mesenchymal markers in the glioblastoma cell lines at the mRNA level, although, at the protein level, this decrease only occurred in the cell lines with more evident mesenchymal phenotypes: LN405 and T98G. However, the inhibition of HDAC6 activity resulted in an increase in acetylated α-tubulin in both cell lines, decreasing their migratory potential. Cell migration is increased by the EMT process, leading to a mesenchymal cellular status and inducing a decrease in acetylated α-tubulin, a protein that might be considered as an epithelial marker, as suggested by Gu, S et al. [60]. Thus, in our study, although there was no decrease in mesenchymal markers after silencing of HDAC6 in U87MG cells, there was an increase in the acetylated α-tubulin epithelial marker, corresponding that fact to a reversion of EMT—or to an activation of the MET (mesenchymal-to-epithelial) process. Therefore, HDAC6 induces EMT in glioblastoma.

The primary cilium appears to be a tumor-suppressor structure in glioblastoma, since normal astrocytes have a perfectly formed primary cilium; but it is absent or aberrantly formed in glioblastoma cells [6,37]. HDAC6 silencing also induced the appearance of the primary cilium in a subpopulation of glioblastoma cell lines, demonstrating the relationship between HDAC6 and the primary cilium. This fact was also demonstrated in cholangiocarcinoma [61], where cells that presented overexpression of HDAC6 did not present a primary cilium, but the ciliary structure was recovered after HDAC6 inhibition. This fact could be related to the decrease in cell proliferation when HDAC6 activity is inhibited, since the presence of the primary cilium could be restored, which, in turn, could regain control of the cell cycle. Treatment with TGFβ induces EMT in kidney epithelial cells, and in turn, aberrant ciliogenesis and EMT is produced in these cells, thus demonstrating that the primary cilium is present in epithelial cells and is lost in mesenchymal cells [62], which is consistent with our results. HDAC6 decreases the expression of mesenchymal markers and, in turn, causes a subpopulation of cells to show a structure similar to the primary cilium. However, the primary cilium is not always found in epithelial cells; in breast cancer, it has been observed in Slug-expressing cells [63], with Slug being an inducer of EMT. So, the primary cilium-EMT relationship is a field yet to be discovered.

Despite having observed Gli1 overexpression in our three glioblastoma cell lines, the Shh pathway was active only in U87MG cells. Therefore, we decided to study the effect of HDAC6 silencing on the Shh pathway in this cell line. HDAC6 silencing decreased the level of Gli1 mRNA expression, which resulted in a reduction in the Shh pathway. Our results corroborate those of Yang et al. [64] in the sense that Shh inhibition was produced after HDAC6 silencing, as they demonstrated that HDAC6 was upregulated in glioma stem cells compared to non-stem tumor cells, and that HDAC6 inhibition downregulated Gli1, Ptch1, and Ptch2 expression and activity in glioma stem cells. A decrease in the Shh pathway decreases cell migration and reverses EMT in lung cancer [65] and gastric cancer [47]. We have also demonstrated these effects after HDAC6 silencing in glioblastoma. These data agree with the decrease in cell viability, but, since the Shh pathway is dependent on the primary cilium, we do not understand yet why it is active in basal conditions despite not having a well-formed primary cilium.

Lastly, we observed that after the silencing of HDAC6, the expression of the autophagy marker p62 and the accumulation of LC3BII also decreased after bafilomycin A1 treatment. These data indicate less autophagy when HDAC6 activity was decreased. In addition, the number of autophagosomes formed in cells after HDAC6 silencing was lower than that of cells treated with siCTRL when autophagy inhibition had been performed by treatment with bafilomycin A1 both under normal conditions and in the absence of nutrients. These data are partially corroborated by Lee J.Y. et al. [66], who conclude that HDAC6 is required for autophagosome-lysosome fusion, and that HDAC6 is not involved in EBSS-mediated autophagy. On the contrary, we show as novel results of our study that silencing of HDAC6 decreases the number of accumulated autophagosomes and not their fusion, and that HDAC6 does intervene in EBSS-mediated autophagy.

From these results, we can assume that HDAC6 fulfills several functions in the development and growth of glioblastoma. This protein is involved in cell proliferation and clonogenicity as well as in the migratory capacity of tumor cells, increased after deacetylation of α-tubulin. We have also observed that HDAC6 inhibition decreases the levels of EMT-TFs Snail and Slug in mesenchymal glioblastoma cell lines and increases the expression of acetylated α-tubulin, the target of HDAC6 and possible epithelial marker, in the three cell lines used in the study. The increased expression of acetylated α-tubulin after HDAC6 inhibition resulted in the appearance of structures similar to the primary cilium in glioblastoma cells, structures that are not present at baseline. Furthermore, the primary cilium is related to a more epithelial-cell phenotype in various tumors. HDAC6 silencing also decreased the activation of the Shh pathway in U87MG cells, the only cell line in which the Shh was active under baseline conditions. Finally, we observed that HDAC6 promotes the autophagic process and decreases it after its inhibition with the mixture of HDAC6 siRNAs. We thus believe that, after the decrease in autophagy, cells cannot obtain all the energy they need for cell growth and proliferation, and, therefore, HDAC6 inhibition has a tumor-suppressing function.

We have demonstrated HDAC6 is involved for the proliferation and migration of GBM cells by the negative regulation of primary cilium formation. This could also be considered as if HDAC6 might regulate differentiation of GBM cells. But our experiments cannot answer that question so far. They represent only an initial point from which to advance further to solve the question. More research is needed to finally figure out a molecular mechanism that regulates the primary cilium formation/differentiation by HDAC6 in glioblastoma cells.

Noorani et al. [67] demonstrated, by means of PiggyBac mutagenesis [68] and exome sequencing, the targets of EGFR amplification or EGFRvIII in gliomas. HDAC6 was not among those targets. As our study has been done with glioblastoma cell lines that express HDAC6, we can estimate that the results obtained would be of interest when applied to glioblastomas that present non-mutated EFGR. But we can also think the opposite: that our results would even be valid for mutant EGFR glioblastomas, as it has been shown that HDAC6-selective inhibitors block activation of the EGFR and p53 pathways by increasing the levels of MSH2 and MSH6, key DNA mismatch repair proteins; by decreasing MGMT expression; and by increasing temozolomide sensitivity and efficiently inducing apoptosis in temozolomide-resistant glioblastoma cells [69]. Certainly, these cells showed higher expression of HDAC6 and more activation of EGFR [26]. HDAC6 inhibitors decreased EGFR protein levels and impaired the activation of the EGFR pathway [26]. Therefore, it is evident that we cannot only rely on PiggyBac results but put them in context with other studies of association of expression of a low number of genes of interest in a given type of tumor.

About the concept of essentiality of genes, we have reports that compare different knock-down expression techniques. Dempster et al. [70] analyzed data from recently published pan-cancer CRISPR-Cas9 screens performed at the Broad and Sanger Institutes and found that the screen results were highly concordant across multiple metrics despite significant differences in experimental protocols and reagents. HDAC6 did not appear as an essential gene in this study. Morgens et al. [71] compared the ability of short hairpin RNA (shRNA) and CRISPR/Cas9 screens to identify essential genes in the human chronic myelogenous leukemia cell line K562. They found that the precision of the two libraries in detecting essential genes was similar and that combining data from both screens improved performance. Genes found uniquely in either the shRNA or the Cas9 screen, but not found in the combination analysis, did not have key signatures of essential genes. In Morgens’ study, HDAC6 appeared to be targeted in Cas9 and shRNA screens, although it could not be validated. But HDAC6 was not targeted in the combo study. Therefore, the results do not clearly show that HDAC6 could be defined under these techniques as an essential gene.

Essential genes have been defined as those required for the survival of an organism or a cell [72]. Some of the essential genes, however, appear to perform non-essential functions, such as aging and cell death, while many of the non-essential genes play critical roles in cell survival. The levels of essentiality of the *Saccharomyces cerevisiae* genes and grouped them into four categories [72]: from conditional essential, in which essentiality is only defined under certain circumstances or growth conditions, to absolute essential, as those genes required for maintaining cell life under a stress-free environment. 

HDAC6 might be, having taken all these notes into account, an example of a non-essential, or at best, of a conditional essential, gene that plays a role in cell survival and proliferation. It is also important to show that HDAC6, far from being an essential gene in its entirety, could be an essential gene for some specific functions, as has been shown in inflammatory breast cancer [72]. The impact of siHDAC6 on glioblastoma cell proliferation is relatively weak, albeit statistically significant. It is therefore unclear how our results might translate into an in vivo setting. Although we do not include in vivo experiments, testing HDAC6 knock-down in mouse experiments in future studies is important, e.g., using mouse models of glioblastoma as those recently reviewed by Noorani [73], who discusses on more faithful mouse models resembling human gliomas, including new cre/LoxP transgenic lines that allow more accurate cell targeting of genetic recombination, Sleeping Beauty [74], and PiggyBac transposons [68] for the integration of transgenes and genetic screens and CRISPR-cas9 [75] for generating genetic knockout and functional screens. Applications of these technologies are providing novel insights into the functional genetic drivers of gliomagenesis, how these genes cooperate with one another, and the potential cells-of-origin of gliomas, knowledge of which is critical to the development of targeted treatments for patients in the clinic [73].

## 5. Conclusions

HDAC6, as well as mesenchymal and autophagic markers, are overexpressed in glioblastoma samples and cell lines. Glioblastoma cell lines lack a primary cilium and present increased autophagy compared with normal human astrocytes. HDAC6 silencing decreases cell proliferation, clonogenicity, and migration in glioblastoma cell lines; restores the primary cilium, reverts the EMT phenotype, and inhibits autophagy; and also inhibits the Shh pathway in U87MG cells. As a whole, we may conclude that HDAC6 seems to be a good target for glioblastoma treatment.

## Figures and Tables

**Table 1:** Sequence of primers used for RT-qPCR.

Primers for RT-qPCR		
Primer Name	Sequence (5′–3′)	Tm (°C)
18S Fw	GTAACCCGTTGAACCCATT	63
18S Rv	CCATCCAATCGGTAGTAGCG	
HDAC6 Fw	GGCTTCAGTTTCCTGTGCTC	63
HDAC6 Rv	TCCTCCATGTTGTCCCTCTC	
Gli1 Fw	AAGCGTGAGCCTGAATCTGT	61
Gli1 Rv	AGCATGTACTGGGCTTTGA	
Snail Fw	GGTTCTTCTGCGCTACTGCT	63
Snail Rv	TAGGGCTGCTGGAAGGTAAA	
Slug Fw	CATTTCAACGCCTCCAAAA	63
Slug Rv	GGAATGGAGCAGCGGTAGT	
P62 Fw	CACTACCGCGATGAGGAC	63
P62 Rv	CTTGTAGCGGGTTCCTACCA	
N-Cadherin Fw	CAGTATCCGGTCCGATCTGC	63
N-Cadherin Rv	AGCTCAAGGACCCAGCAGTG	

## Data Availability

Not applicable.

## Supplementary Materials

The following are available online at https://www.mdpi.com/article/10.3390/biology10060467/s1, Figure S1: HDAC6 silencing decreases the number of autophagosomes per cell in T98G and U87MG cell lines. (A) Representative image of autophagosomes per cell of T98G cell line after siRNAs treatment for 72 h and 100 nM Baf A1, EBSS, EBSS + Baf A1, or DMSO (as vehicle control) treatment for 6 h. (B) Representative image of autophagosome per cell of U87MG cell line after siRNAs treatment for 72 h and 100 nM Baf A1, EBSS, EBSS + Baf A1, or DMSO (as vehicle control) treatment for 6 h.

## Abbreviations

AGM	Astrocyte growth medium
ATP	Adenosine triphosphate
cDNA	Complementary DNA
DMSO	Dimethyl sulfoxide
EBSS	Earle’s balanced salt solution
EGFR	Epidermal growth factor receptor
EMT	Epithelial-mesenchymal transition
EMT-TF	Epithelial-mesenchymal transition transcription factor
FBS	Fetal bovine serum
GBM	Glioblastoma multiforme
HDAC	Histone deacetylase
HSP90	Heat shock protein 90
MET	Mesenchymal-epithelial transition
MGMT	O6-methylguanine DNA methyltransferase
P/S	Penicillin and streptomycin
PBS	Phosphate-buffered saline
PCR	Polymerase chain reaction
Ptch	Patched
RPMI	Roswell Park Memorial Institute
RT	Reverse transcriptase
Shh	Sonic Hedgehog
TGFβ	Transforming growth factor beta
