# Supplementary figures and images for: Silencing of Histone Deacetylase 6 Decreases Cellular Malignancy and Contributes to Primary Cilium Restoration, Epithelial-to-Mesenchymal Transition Reversion, and Autophagy Inhibition in Glioblastoma Cell Lines
Source: Biology (Basel). 2021 May 26;10(6):467. doi: 10.3390/biology10060467 (PMC8228543; doi:10.3390/biology10060467)

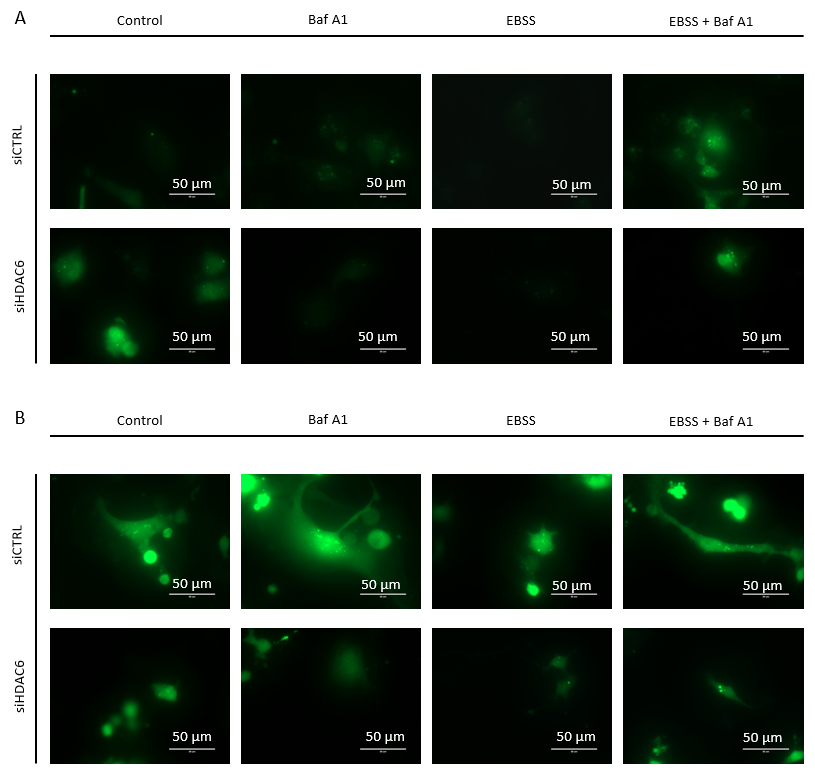

Supplement: Supplementary file 1 [file biology-10-00467-s001.zip › biology-1214777-supplementary/Figure S1.png]
